# Diversity and Ecological Guild Analysis of the Oil Palm Fungal Microbiome Across Root, Rhizosphere, and Soil Compartments

**DOI:** 10.3389/fmicb.2022.792928

**Published:** 2022-02-11

**Authors:** Eleanor R. Kirkman, Sally Hilton, Gomathy Sethuraman, Dafydd M. O. Elias, Andrew Taylor, John Clarkson, Aik Chin Soh, David Bass, Gin Teng Ooi, Niall P. McNamara, Gary D. Bending

**Affiliations:** ^1^School of Life Sciences, University of Warwick, Coventry, United Kingdom; ^2^Crops for the Future Research Centre, Semenyih, Malaysia; ^3^Faculty of Science, Institute of Biological Sciences, University of Malaya, Kuala Lumpur, Malaysia; ^4^UK Centre for Ecology & Hydrology, Lancaster Environment Centre, Bailrigg, United Kingdom; ^5^Department of Life Sciences, Natural History Museum, London, United Kingdom; ^6^Centre for Environment, Fisheries and Aquaculture Science, Weymouth, United Kingdom

**Keywords:** oil palm, rhizosphere, root, fungi, pathogen, arbuscular mycorrhizal fungi, tropical

## Abstract

The rhizosphere microbiome is a major determinant of plant health, which can interact with the host directly and indirectly to promote or suppress productivity. Oil palm is one of the world’s most important crops, constituting over a third of global vegetable oil production. Currently there is little understanding of the oil palm microbiome and its contribution to plant health and productivity, with existing knowledge based almost entirely on culture dependent studies. We investigated the diversity and composition of the oil palm fungal microbiome in the bulk soil, rhizosphere soil, and roots of 2-, 18-, and 35-year old plantations in Selangor, Malaysia. The fungal community showed substantial variation between the plantations, accounting for 19.7% of community composition, with compartment (root, rhizosphere soil, and bulk soil), and soil properties (pH, C, N, and P) contributing 6.5 and 7.2% of community variation, respectively. Rhizosphere soil and roots supported distinct communities compared to the bulk soil, with significant enrichment of Agaricomycetes, Glomeromycetes, and Lecanoromycetes in roots. Several putative plant pathogens were abundant in roots in all the plantations, including taxa related to *Prospodicola mexicana* and *Pleurostoma* sp. The mycorrhizal status and dependency of oil palm has yet to be established, and using 18S rRNA primers we found considerable between-site variation in Glomeromycotinian community composition, accounting for 31.2% of variation. There was evidence for the selection of Glomeromycotinian communities in oil palm roots in the older plantations but compartment had a weak effect on community composition, accounting for 3.9% of variation, while soil variables accounted for 9% of community variation. While diverse Mucoromycotinian fungi were detected, they showed very low abundance and diversity within roots compared to bulk soil, and were not closely related to taxa which have been linked to fine root endophyte mycorrhizal morphology. Many of the fungal sequences showed low similarity to established genera, indicating the presence of substantial novel diversity with significance for plant health within the oil palm microbiome.

## Introduction

Palm oil is a versatile vegetable oil derived from the fruit of oil palm trees *Elaeis guineensis* (Sambanthamurthi et al., 2000), and comprises ≈36% of total worldwide vegetable oil production. Global demand for vegetable oils and biofuels is expected to upsurge in the forthcoming years, with biodiesel requirements reaching ≈277 Mt, and edible oil demands escalating to ≈240 Mt by 2050 (Koh, 2007; Corley, 2009). Malaysia and Indonesia are currently the largest producers of palm oil, encompassing ≈85% of worldwide production (Mukherjee and Sovacool, 2014). Oil palm cultivation areas in Malaysia increased dramatically from 54,000 hectares in 1960 to 5.8 million hectares (Mha) in 2018 (Basiron, 2007; The World Bank, 2020).

Oil palm is a particularly appealing crop for biofuel, food, and chemical production due to its higher oil yield per hectare relative to other oil producing crops (Murphy, 2014). For example, soybean oil is the second most consumed vegetable oil worldwide but requires 10 times the land area of oil palm (≈90 Mha) to produce an equivalent oil yield, highlighting the economic and livelihood benefits of oil palm cultivation (Basiron, 2007). However, it is widely recognized that land clearing of native tropical forest in Southeast Asia has had devastating environmental consequences, particularly for biodiversity and carbon storage. Substituting palm oil with alternative vegetable oil crops could create new environmental consequences following land clearance if expanded into other regions of the world (Mejaard et al., 2018). Therefore, improving productivity in existing oil palm lands is vital for future sustainable vegetable oil supply.

Plant roots live in association with diverse microbial communities recruited from the soil, with environmental factors and plant characteristics such as age and genotype influencing diversity and composition (Hunter et al., 2015). The rhizosphere microbiome has major influences on plant health, growth and productivity (Bennett et al., 2012). This occurs through diverse pathways, including indirect interactions with free-living microbes which enhance nutrient availability for the plant, and direct interactions with pathogens and mutualists (Morgan et al., 2005). There is considerable interest in devising biotechnological approaches to engineer crop microbiomes for beneficial traits, such as disease suppression and nutrient mobilization for the sustainable improvement of crop health and yield (Ryan et al., 2009).

Studies of plant associated microbial communities of oil palm have predominantly focused on the use of culturing to isolate rhizosphere bacteria and investigate their potential for promoting plant growth. This has included bacterial phosphate solubilization (Acevedo et al., 2014) and antagonism of pathogens. Particular focus has been on investigating the potential of rhizosphere bacteria to control the white rot fungus *Ganoderma boninense*, which causes the widespread and economically damaging basal stem rot in oil palm plantations (Nur Azura et al., 2016; Shariffah-Muzaimah et al., 2017). Furthermore, oil palm has the capacity to form arbuscular mycorrhizal symbioses and there has been recognition of the role that these fungi could play in sustainable oil palm cultivation (Phosri et al., 2010). Studies have indicated that inoculation of oil palm seedlings with arbuscular mycorrhizal fungi (AMF) has potential to control *G. boninense* infection (Sundram et al., 2015) and promote plant growth and phosphorus uptake (Blal et al., 1990; Galindo-Castañeda and Romero, 2013). However, the mycorrhizal status and dependency of oil palm grown in plantations remains unclear, with recent evidence suggesting low colonization of roots by AMF in monoculture oil palm plantations in Amazonia (da Silva Maia et al., 2021).

Development of next generation sequencing approaches has provided opportunities for culture-independent analysis of microbiomes for fine-level resolution of the diversity and composition of plant-associated microbiomes, characterization of the factors which shape community composition, and to reveal microbial interactions which affect plant health (Hilton et al., 2021). Several studies have used culture-independent approaches to investigate how plantation management (Ballauff et al., 2020) and the conversion of native forest to oil palm cultivation impacts the soil microbiome. Soil bacterial and fungal communities are impacted by oil palm cultivation (Lee-Cruz et al., 2013; Tripathi et al., 2016), including reduced abundance of ectomycorrhizal fungal associates of dipterocarp trees (McGuire et al., 2015), and increased abundance of AMF (Krashevska et al., 2015; Sahner et al., 2015). Furthermore, diversity of oil palm phyllosphere communities has identified a range of putative pathogens associated with both asymptomatic plants, and plants showing symptoms of fatal yellowing disease, the cause of which remains unknown (de Assis Costa et al., 2018).

Oil palm starts producing fruit approximately 2–3 years after planting, maintaining stable productivity for approximately 15 years, when yields gradually decline. Plantations are typically replanted after 25–30 years, when the canopy becomes too high for harvesting, or the crops have become affected by pests and diseases (Woittiez et al., 2017). Plantation age could thus be a key driver of community assembly, reflecting both land use legacy and temporal changes in biodiversity with direct impacts on plant health.

In this study, we investigated determinants of the composition of the belowground fungal microbiome of oil plalm with particular focus on putative pathogens and AMF. Samples from soil, rhizosphere soil and root compartments were collected from a 2-, an 18-, and a 35-year old plantation growing within close geographical proximity in Selangor, Malaysia. Internal transcribed spacer (ITS) sequencing was used to investigate the relative importance of plantation site, compartment and soil properties (pH, C, N, and P) for determining fungal community composition. This data was also used to investigate composition and diversity of fungal ecological guilds. AMF communities can comprise both Mucoromycotinian and Glomeromycotinian fungi, and detailed analysis of the diversity and composition of these groups was conducted using 18S rRNA sequencing. The relative importance of plantation, compartment and soil properties for shaping Glomeromycotinian fungal community composition was determined.

## Materials and Methods

### Site Description, Sample Collection, and Soil Analysis

**FIGURE 1:**
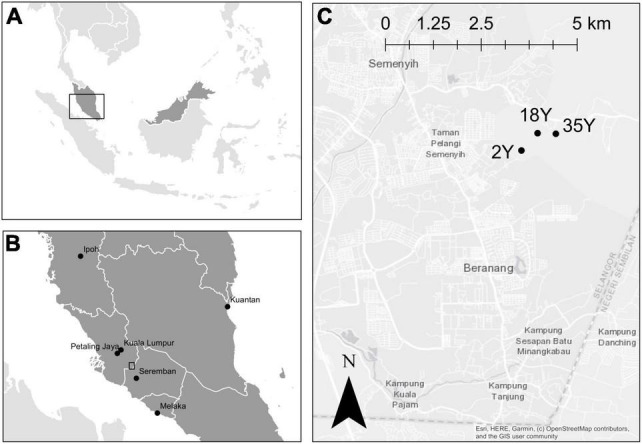
Location of 2-, 18-, and 35-year old oil palm plantations at Selangor on Peninsular Malaysia. **(A)** Location of the study area is indicated by a box. **(B)** Regional location, sampling area indicated by a box. **(C)** Spatial arrangement of plantations at Selangor.

Soil and root samples were collected on February 27th 2017 from the Crops for the Future Research Centre at Selangor in Peninsular Malaysia (Figure 1). This center covers 12.8 Ha and forms part of the wider Balau Estate (Boustead Plantations Berhad), which is mainly planted with oil palm. The climate is tropical and aseasonal with an average annual temperature of 27.2°C. Eleven years mean annual rainfall ranges from 1,454 to 2,808 mm with a mean of 1,987 mm. Three oil palm plantations (35-year old, 10 ha area, 2°56 05.4N 101°52 59.6E; 18-year old, 10 ha area, 2°56 05.9N 101°52 44.2E; 2-year old, 8 ha area, 2°55 51.2N 101°52 30.5E) were selected for sampling. The 35-year old plantation was 0.8 km from the 18-year old plantation and 1.7 km from the 2-year old plantation, while the 2-year old plantation was 1 km from the 18-year old plantation. The 18- and 35-year old sites were second rotation plantations, while the 2-year old site had previously been an oil palm nursery. The understorey in 18- and 35-year old plantations was treated with glufosinate ammonium twice annually. Two-year old palms were fertilized at 4 monthly intervals with Nitrophoska blue (N:P:K:Mg – 12:12:17:2) at a rate of 2 kg per palm. The palm circle was treated with glufosinate ammonium at 3 monthly intervals. The soils were sandy-clay loams (73% sand, 5% silt, and 22% clay) from the Rengam Malaysian soil series derived from acid igneous parent material.

In each plantation, samples were collected from trees at nine equally spaced positions 12 m apart along a “W” shaped sampling pattern. At each sampling position a hole was excavated 1 m from the tree, within the palm circle, to 20 cm depth and approximately 10 g of oil palm roots and 200 g soil were collected into zip lock bags. Materials were stored at 4°C and DNA was extracted within 24 h.

To obtain separate bulk soil, rhizosphere soil and root samples for DNA extraction the method of Hilton et al. (2021) was followed. Loosely adhering soil was removed from the roots by gentle shaking to leave closely adhering soil. 6 g of roots were sequentially washed vigorously in 4 × 25 ml washes of sterile distilled water. The soil removed by washing was collected by centrifugation (3,250 × *g* for 10 min) and retained as rhizosphere soil. The washed roots were visually examined and dead and senescent roots, or roots which lacked turgor, were removed. The remaining roots were cut into 5 mm pieces. Bulk soil was sieved first through 7 mm and then 2 mm sieves and 6 g was washed in sterile distilled water using the same sequential washing technique as used for collection of the rhizosphere soil samples. Sieved bulk soil samples were analyzed for total carbon and nitrogen, extractable phosphorus (Olsen-P) and pH using protocols outlined in Barnes et al. (2018).

### DNA Extraction and Sequencing

Root, rhizosphere soil, and bulk soil samples of 500 mg were extracted according to the manufacturer’s instructions using the Exgene Soil DNA kit (Cambio), with the exception that the samples were homogenized in a TissueLyser II (Qiagen) at 20 Hz for 2 × 4 min with a 180° rotation of the plates between homogenizations. DNA yield was increased by adding 1/4 inch ceramic sphere (MP Biomedicals) to extraction tubes.

For each sample, the fungal ITS2 region (fITS7-ITS4, Strid et al., 2012) was used to amplify the fungal metacommunity, and the arbuscular mycorrhizal fungus (AMF) community was amplified using 18S rRNA gene primers AMV4.5NF and AMDGR (Sato et al., 2005) which amplifies both Glomeromycotinian and Mucoromycotinian fungi (Orchard et al., 2017). The primer sets were modified at the 5′ end with adapters from a dual-index sequencing strategy (Kozich et al., 2013). PCR reactions were performed using 15 ng of DNA, Q5^®^ Hot Start High-Fidelity 2X Master Mix (New England Biolabs) and 0.5 mM of each primer, in a reaction volume of 25 ml. SequelPrepTM Normalization Plate Kit (Invitrogen) was used to purify amplicons. The Illumina MiSeq Reagent Kit v3 (2 × 300-cycle) was used to sequence the libraries. The fungal ITS and 18S rRNA datasets described in this article are available in the NCBI sequence read archive under BioProject ID PRJNA755964 and PRJNA756009, respectively.

Trimmomatic v0.35 (Bolger et al., 2014) was used after sequencing to remove low-quality bases from the sequence ends. The following steps were performed using USEARCH and UPARSE software (Edgar, 2010, 2013). The forward and reverse reads were aligned and quality filtered (-fastq_maxee 0.5) to assemble paired-ends reads. Singletons and chimeric sequences were discarded from the dataset and unique sequences were arranged according to their abundance. Sequences were clustered to OTUs at 97% minimum identity threshold (usearch-cluster_otus), where chimeras are removed using chimera filters integrated into the algorithm. Further chimeras were removed using uchime-ref and the databases used for taxonomy assignment. Quantitative Insights into Microbial Ecology (QIIME 1.8, Caporaso et al., 2010) was used to assign taxonomy to OTUs, with the UNITE v8.2 database used for ITS (Koljalg et al., 2013), and the SILVA database (Quast et al., 2013) used for 18S rRNA.

A total of 2.63 million sequences were obtained in the ITS dataset, while 2.52 million 18S rRNA sequences were obtained. Only sequences belonging to the Glomeromycotina (63.3% of reads) and Mucoromycotina (1.2% of reads) were retained in the 18S rRNA dataset. Operational taxonomic unit (OTU) tables were rarefied to 20,000 and 10,000 sequences per sample for the ITS and 18S rRNA sequences, respectively (Supplementary Figure 1). This produced 4878 ITS OTUs, and for the 18S rRNA data 607 Glomeromycotina and 100 Mucoromycotina OTUs.

### Fungal Guild Analysis

ITS OTUs were allocated to pathotroph, symbiotroph and saprotroph trophic modes, and plant pathogen, ectomycorrhizal (EcM) fungus and AMF guilds using the FUNGuild v1.0 tool (Nguyen et al., 2016). OTUs assigned a multitrophic identity (i.e., more than one trophic mode) were not included. Only OTUs with confidence rankings of “highly probable” and “probable” were included.

### Statistical Analyses

The vegan (Oksanen et al., 2018) package in R was used to calculate Bray-Curtis dissimilarities for amplicon data, and these were visualized using non-metric multidimensional scaling (NMDS), and plotted using ggplot2 (Wickham, 2016). ggtern in R (Hamilton and Ferry, 2018) was used to create ternary plots. PERMANOVA was conducted using the vegan package to investigate the importance of site, compartment (i.e., bulk soil, rhizosphere soil, and roots) and soil variables in contributing to community composition. The Kruskal–Wallis rank sum test was used to determine significant differences in soil properties between plantations, and to investigate differences in alpha diversity and fungal guilds between plantations and compartments. A Dunn’s test using the FDR with the Benjamini-Hochberg procedure was used to correct *P*-values for multiple comparisons. Similarity percentage analysis (SIMPER) was conducted in the PAST4 program (Hammer et al., 2001) to determine the contributions of specific OTUs to the observed differences in microbial community structure between plantations and compartments.

### Phylogenetic Analysis

Similarity percentage analysis identified a number of ITS OTU which were highly enriched in roots relative to bulk soil, but which had low homology to GenBank sequences. To obtain more detailed information about the phylogenetic relatedness of these sequences, the most closely related sequences to these OTUs were downloaded from NCBI GenBank, and sequence alignments were generated using MAFFT v.7 (Katoh and Standley, 2013) (E-INS-i algorithm). Phylogenetic analyses were performed on the CIPRES Science Gateway. Maximum likelihood analyses were performed with RAxML v8 (Stamatakis, 2014). Phylogenetic analysis of Mucoromycotina 18S rRNA gene sequences was performed to investigate relatedness to taxa which are considered to form the fine root endophyte arbuscular symbiosis. Reference sequences of putative Mucoromycotinian arbuscular mycorrhizal fungi (M-AMF) were obtained from Orchard et al. (2017) and Albornoz et al. (2021), and these were supplemented with a broad range of Mucoromycotinian sequences which encompassed the known biodiversity within this group. Sequence alignments were generated with the MUSCLE alignment tool using default parameters (Edgar, 2004). Evolutionary history was inferred using the Maximum Likelihood method and Kimura 2-parameter mode with 1000 bootstraps. The tree with the highest log likelihood was selected. A discrete Gamma distribution was used to model evolutionary rate differences among nucleotide sites. All positions with less than 95% site coverage were eliminated. There were a total of 206 positions in the final dataset. Evolutionary analyses were conducted in MEGA X (Kumar et al., 2018).

## Results

### Plantation Soil Characteristics

Soil carbon, nitrogen and pH were all significantly lower (*P* < 0.05) at the 2 years plantation relative to the 18- and 35-year old plantations, in which soil properties were not significantly different (Supplementary Figure 2). Amounts of Olsen-P were significantly higher (*P* < 0.05) in the 18-year old plantation relative to the 2- and 35-year old sites, which were not significantly different to each other.

### Fungal Community Composition Across Sites and Compartments

**FIGURE 2:**
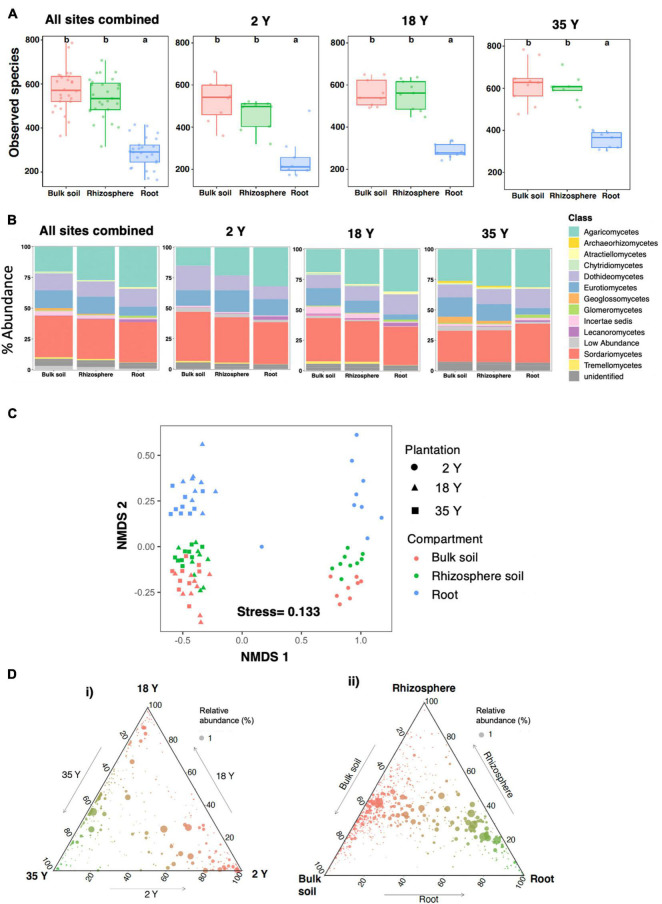
Fungal community characteristics in soil, bulk soil and rhizosphere compartments in 2-, 18-, and 35-year old oil palm plantations. Sequencing was performed using ITS primers. **(A)** Alpha diversity of fungal OTUs. Bars with different letters are significantly different (*p* < 0.05). Error bars represent the minimum and maximum values, excluding outliers. **(B)** Stacked bar charts showing the relative abundance of fungal classes. **(C)** Non-metric multidimensional scaling analysis of Bray Curtis dissimilarity of fungus communities at the OTU level. **(D)** Ternary plots showing distribution of fungal OTU across **(i)** oil palm plantations (bulk soil, rhizosphere soil and roots combined at each site) and **(ii)** bulk soil, rhizosphere soil and root compartments (plantations combined for each compartment).

Across all sites combined, alpha diversity determined using ITS sequencing was not significantly different between the bulk soil and rhizosphere soil (Figure 2A). However, diversity in roots was approximately half that in the bulk soil and rhizosphere soil (significant *P* < 0.05). Similar trends were seen when each site was analyzed separately.

Analysis of fungal community composition at the class level revealed that when data from all sites was combined, with progression from bulk soil, through to rhizosphere soil and root there was significant (*P* < 0.05) enrichment in the relative abundance of Agaricomycetes, Glomeromycetes and Lecanoromycetes and reduced relative abundance of Eurotiomycetes, Geoglossomycetes, and Tremellomycetes (Figure 2B). Analysis of each plantation separately showed similar trends.

When data from all three plantations was combined, PERMANOVA (Table 1) and NMDS (Figure 2C) analysis showed that the main factors determining fungal community composition were plantation and compartment, which accounted for 19.7% (significant *P* < 0.001) and 6.5% of community variation (significant *P* < 0.001), respectively. Soil pH, C, N, and P accounted for 2.2, 1.8, 1.6, and 1.6% of community variation, respectively (significant *P* < 0.003, 0.008, 0.008, and 0.023, respectively). Visual analysis of the NMDS plot showed close clustering of compartments between the 18- and 35-year old plantations, which were distinct from those of the 2-year old plantation. Root communities were clearly separated from bulk soil and rhizosphere soil communities in each plantation.

**TABLE 1:** Permutational multivariate analysis of variance (PERMANOVA) of the effect of plantation (2-, 18-, and 35-year old locations), compartment (soil vs. rhizosphere soil vs. root) and soil characteristics on fungal community composition.

	DF	SS	MS	*F*.Model	*R* ^2^	Pr(>F)
Plantation	2	5.925	2.963	10.515	0.197	0.001
Compartment	2	1.94	0.972	3.451	0.065	0.001
pH	1	0.675	0.673	2.397	0.022	0.003
Soil C	1	0.536	0.536	1.904	0.018	0.008
Soil N	1	0.484	0.484	1.718	0.016	0.008
Olsen P	1	0.467	0.467	1.657	0.016	0.023
Residuals	71	20.005	0.282		0.666	
Total	79	30.038			1.000	

*Sequencing was performed using ITS primers.*

Ternary plots indicated that a substantial proportion of OTUs were shared between the 18- and 35-year old plantations (Figure 2Di), with a distinct community in the 2-year old plantation which was absent or present in low relative abundance at the other sites. Analysis of OTU distribution across compartments (Figure 2Dii) showed that a substantive proportion of OTUs were selectively enriched in the rhizosphere and root compartments, although there was a significant community, including several highly abundant OTU which were equally abundant across all compartments.

Similarity percentage analysis identified taxa which contributed to the dissimilarity in community composition between the root, rhizosphere soil and bulk soil compartments, when data from all three plantations was combined (Table 2). This identified a number of ascomycete and basidiomycete OTUs which were enriched in the root relative to the bulk soil and rhizosphere soil compartments. The Hypocreales OTU0 had 5.8% relative abundance in roots compared to 0.8% in soil and contributed the most dissimilarity between compartments (2.5%). Phylogenetic analysis suggested that this OTU is most closely related to the plant pathogen *Pleurostoma richardsiae* (Supplementary Figure 3). OTU6 contributed 2.4% to dissimilarity in community composition between compartments and comprised 4.9% of fungal relative abundance in roots compared to 0.9% in soil. This OTU was most closely related to *Tubulicium spp*. (Supplementary Figure 3), with the closely related sequence KU195513 detected growing on roots of *Allanblackia stuhlmannii* in Tanzania (Fransson et al., 2016), and an epiphyte in Costa Rica (Kartzinel et al., 2013).

**TABLE 2:** Similarity percentage (SIMPER) analysis of OTU contributing to fungus community dissimilarity between root, rhizosphere soil, and bulk soil compartments.

OTU	% contribution to difference	% relative abundance in bulk soil	% relative abundance in rhizosphere soil	% relative abundance in roots
Hypocreales OTU0	2.5	0.8	1.9	5.8
*Tubulicium raphidisporum* OTU6	2.4	0.9	2.4	4.9
*Prosopidicola mexicana* OTU8	2.3	0.2	1.4	5.3
Dothideomycetes OTU1	2.1	0.6	2.5	3.9
*Talaromyces proteolyticus* OTU2	2.0	3.1	2.6	0.4
*Geastrum morganii* OTU12	1.9	2.5	1.9	0.3
Agaricomycetes OTU3	1.8	0.8	1.7	3.7
Dothideomycetes OTU4	1.8	0.4	1.1	3.6
*Phallus* sp. OTU5	1.8	0.9	2.3	1.7
*Clavulinopsis luteonana* OTU13	1.4	0.5	1.1	2.1
Sordariomycetes OTU10	1.3	0.8	1.5	1.7
*Ramaria* sp. OTU20	1.2	0.2	1.3	1.7
*Talaromyces francoae* OTU7	1.2	1.6	1.9	1.4
Atheliaceae OTU11	1.1	0.2	0.8	2.3
*Phallus atrovolvatus* OTU28	1.1	1.2	1.4	0.3

*Sequencing was performed using ITS primers. Data is pooled across 2-, 18-, and 35-year old plantations.*

OTU8 formed 5.3% relative abundance of the root associated fungus community compared to 0.2% of the soil community, and contributed to 2.3% of fungus community dissimilarity across compartments. This fungus was most closely related to the plant pathogen *Prosopidicola mexicana* (Supplementary Figure 3). OTU1 comprised 3.9% of relative abundance in roots compared to 0.6% in soil, and contributed 2.1% of variation in fungal communities across compartments. The sequence was assigned to the Dothideomycetes, with BLAST analysis showing relatedness to the Pleosporales order, and 100% similarity to several sequences detected previously in roots, including JX391947 from date palm roots and FJ752619 from wild rice roots (Yuan et al., 2010).

OTU3 contributed 1.8% to variation in fungal communities across compartments, and formed 3.7% of the fungal relative abundance in roots compared to 0.8% in soil, and was aligned to the polyporales, with closely related sequences previously detected in roots of a tropical tree (KTT224914), the rhizosphere of potato in the Netherlands (HM037681, Hannula et al., 2012) and in tropical soil in Puerto Rica (KT241374, Urbina et al., 2016). This clade was most closely related to *Fomitopsis palustris* (Supplementary Figure 3). OTU4 contributed 1.8% of variation in fungal community composition across compartments, and formed 3.6% of relative abundance in roots, compared to 0.4% in soil. Taxonomic analysis placed OTU4 within the Pezizomycotina, and it showed close similarity to a root associated sequence (JF519577) identified in *Allanblackia stuhlmannii* in Tanzania, in the same root sample as KU195513, which was related to OTU6. The most closely related described species to this clade was *Neolinocarpon rachidis*.

These fungi were also important in determining dissimilarity of fungal community composition between sites (Table 3), contributing between 2.8 and 4% of fungal community dissimilarity. OTUs 0, 6, 4, and 1 comprised between 5.6 and 10.4% of relative abundance in the 18-year old plantation, 5.0–6.3% relative abundance at the 35-year old plantation, but only 0.0–0.7% relative abundance at the 2-year plantation. In contrast OTU3 and 8 had greater relative abundance in the 2-year old plantation (5.9 and 7.4%, respectively) relative to the 35-year old (3.6 and 3.9% relative abundance, respectively), and 18-year old (1.7 and 4.5% relative abundance, respectively) plantations.

**TABLE 3:** Similarity percentage (SIMPER) analysis of OTU contributing to fungus community dissimilarity between 2-, 18-, and 35-year old plantations.

OTU	% contribution to difference	% relative abundance in 2-year old plantation	% relative abundance in 18-year old plantation	% relative abundance in 35-year old plantation
Hypocreales OTU0	4.0	0.7	10.4	6.3
*Tubulicium raphidisporum* OTU6	4.0	0.1	8.8	5.9
*Prosopidicola mexicana* OTU8	3.7	7.4	4.5	3.9
Dothideomycetes OTU4	3.5	0.0	5.6	5.0
Dothideomycetes OTU1	3.2	0.1	6.4	5.4
Agaricomycetes OTU3	2.8	5.9	1.7	3.6
*Clavulinopsis luteonana* OTU13	2.3	0.0	5.0	1.4
Atheliaceae OTU11	2.0	0.1	3.1	3.6
Sordariomycetes OTU10	2.0	4.9	0.1	0.0
*Phallus* sp. OTU5	2.0	0.4	4.2	0.6
*Ramaria* sp. OTU20	2.0	4.2	1.0	0.0
*Endoxyla macrostoma* OTU46	1.5	0.1	0.3	3.5
*Talaromyces francoae* OTU7	1.3	3.3	0.7	0.2
Agaricales OTU54	1.2	0.0	2.8	0.2
*Arcopilus fusiformis* OTU23	1.2	2.8	0.0	0.0
Unidentified OTU64	1.2	2.0	1.0	0.4
*Gymnopus* sp. OTU17	1.1	2.7	0.0	0.0
Basidiomycota OTU15	1.1	0.2	2.2	0.1
Mycenaceae OTU53	1.0	0.2	0.2	2.2
*Marasmiellus griseobrunneus* OTU96	1.0	0.0	1.4	1.1

*Sequencing was performed using ITS primers. Bulk soil, rhizosphere soil and root compartments are pooled within each plantation.*

### Distribution of Fungal Trophic Modes Across Compartments and Between Sites

Of the 4,528 ITS OTUs identified, 2,138 were assigned a trophic mode by FUNGuild under a “probable” or “highly probable” confidence ranking. 46.2% of these OTUs were saprotrophs, 16.9% were pathotrophs, 16.7% were symbiotrophs and the remaining 20.2% were multitrophic. Of the 362 pathotroph OTUs identified, 245 were plant pathogens, while of the 357 symbiotroph OTUs identified, 185 were AMF, 91 ECM fungi and 20 were endophytes. When all 3 plantations were combined (Supplementary Figure 4Ai) the relative abundance of saprotroph OTUs was significantly (*P* < 0.05) lower in the roots (27%) relative to the bulk soil and rhizosphere soil (35%), while the relative abundance of plant pathogens (Supplementary Figure 4Aii) increased significantly (*P* < 0.05) from 4.5 to 5% in the bulk soil and rhizosphere soil to 8.5% in the roots. AMF (Supplementary Figure 4Aiii) increased significantly (*P* < 0.05) from 0.4 to 0.6% in the rhizosphere and bulk soil to 1.6% in the roots. EcM fungi comprised less than 0.5% of relative abundance with no significant difference between compartments. When compartments within each plantation were combined (Supplementary Figure 4Bii), there was significantly (*P* < 0.05) greater relative abundance of plant pathogens in the 2-year old plantation (10%) relative to the 18- and 35-year old plantations (3.8–4.0%), while relative abundance of AMF (Supplementary Figure 4Biii) was significantly (*P* < 0.05) different between plantations (0.5, 1.0, and 1.5% in the 2-, 18-, and 35-year old plantation, respectively).

Further analysis showed that AMF retrieved from the ITS dataset comprised 2.6% of fungus community relative abundance in the roots of the 35-year old plantation, compared to 1.7% in the 18-year old plantation and 0.7% at the 2-year old site (Supplementary Figure 5A). There was no significant difference in AMF or plant pathogen (Supplementary Figure 5B) relative abundance between soil and root compartments in the 2- and 18-year old plantations, but in the 35-year old plantation the relative abundance of both AMF and plant pathogens was significantly higher in the roots relative to the bulk soil and rhizosphere.

Permutational multivariate analysis of variance (Supplementary Table 1) revealed that plantation significantly affected AMF community composition accounting for 24% of community variation, with soil pH, C, N and Olsen P also contributing between 1.9 and 2.6% of community variation. However, compartment had no significant effect on AMF community composition, and NMDS analysis (Supplementary Figure 6A) showed clear clustering of samples by plantation but not compartment. For plant pathogens, plantation and compartment contributed to 18.2% (*P* < 0.001) and 7.0% (*P* < 0.001) of the variation in community composition, respectively (Supplementary Table 2), with NMDS (Supplementary Figure 5B) showing clustering of the 18- and 35-year old plantations together, which were clearly separated from the 2-year old plantation. Roots were clearly separated from bulk soil and rhizosphere soil in all plantations. SIMPER analysis of plant pathogens (Supplementary Table 3) identified OTU8 (*Prosopidicola mexicana*) as contributing to 36.7% of the variance between root and bulk soil compartments, in which it had 5.3 and 0.2% relative abundance, respectively. OTU74 (Magnaporthaceae), 78 (*Acrophialophora levis*), 142 (*Veronaea botryosa*), and 150 (*Cylindrosympodium* sp.) showed evidence for enrichment in roots relative to bulk soil, and contributed to between 5.6 and 2.4% of the variation in plant pathogen community composition between roots and soil. In contrast OTU36 (*Plectosphaerella oligotrophica)*, OTU98 (*Triparticalcar sp.*), OTU141 (*Plectosphaerella delsorboi*) and OTU125 (*Diatrypella atlantica*) contributed 6.1, 4.9, 3.6, and 2.6% of the dissimilarity between root and bulk soil compartments, with greater relative abundance in the soil relative to the root.

### Arbuscular Mycorrhizal Fungi Community Composition Across Sites and Compartments

**FIGURE 3:**
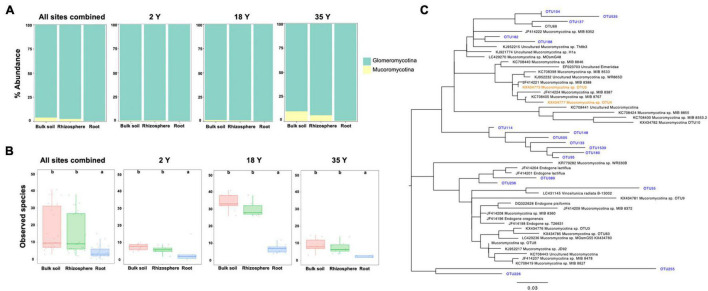
Mucoromycotinian relative abundance, diversity and taxonomic composition in 2-, 18-, and 35-year old plantations. Sequencing was performed with 18S rRNA gene primers. **(A)** Stacked bar chart showing relative abundance of Glomeromycotinian and Mucoromycotinian fungal subphyla. **(B)** Observed species of Mucoromycotinian fungal OTUs. Bars with different letters are significantly different (*p* < 0.05). Error bars represent the minimum and maximum values, excluding outliers. **(C)** Phylogenetic analysis of Mucoromycotinian 18S rRNA gene sequences. OTUs sequenced in this study are shown in blue. Partial 18S rRNA sequence of the putative Mucoromycotina arbuscular mycorrhizal fungus (fine root endophyte) KX43777 (OTU4) and KX43773 (OTU0) from Orchard et al. (2017) are shown in orange.

**FIGURE 4:**
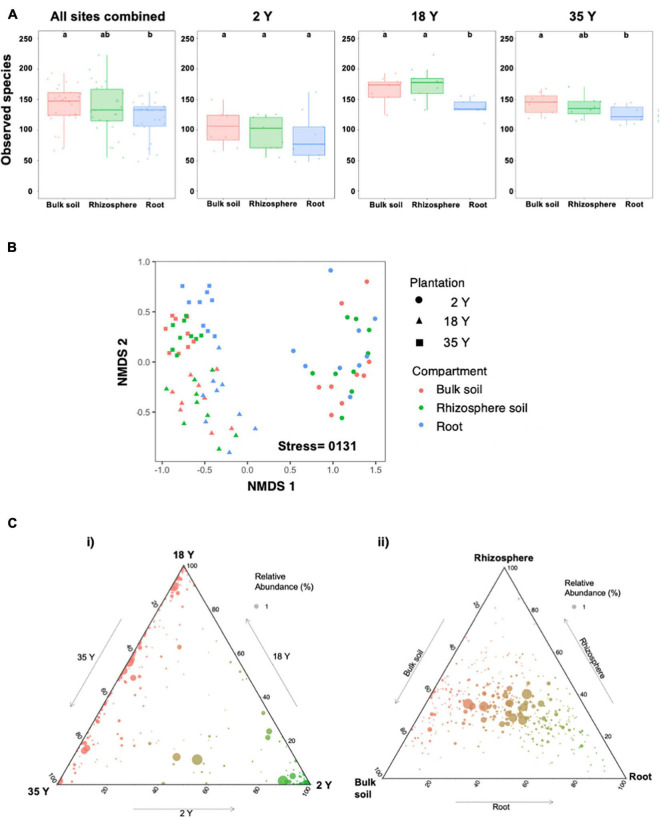
Glomeromycotinian community characteristics in soil, bulk soil and rhizosphere compartments in 2-, 18-, and 35-year old oil palm plantations. Sequencing was performed with 18S rRNA gene primers. **(A)** Alpha diversity of Glomeromycotinian fungus OTUs. Bars with different letters are significantly different (*p* < 0.05). Error bars represent the minimum and maximum values, excluding outliers. **(B)** Non-metric multidimensional scaling analysis of Bray Curtis dissimilarity of Glomeromycotinian communities at the OTU level. **(C)** Ternary plots showing distribution of Glomeromycotinian OTUs across **(i)** oil palm plantations (bulk soil, rhizosphere soil, and roots combined at each site) and **(ii)** bulk soil, rhizosphere soil and root compartments (plantations combined for each compartment).

A more detailed analysis of AMF community composition was conducted using an 18S rRNA primer set. When plantations were combined, Mucoromycotina sequences comprised 4.0, 2.3, and 0.2% of relative abundance in bulk soil, rhizosphere soil, and roots, respectively (Figure 3A). This trend for reduced relative abundance in roots occurred across all plantations. When all 3 plantations were combined both Mucoromycotinian (Figure 3B) and Glomeromycotinian (Figure 4A) OTU richness was significantly lower in roots relative to the bulk soil, and similar trends occurred in all plantations.

Of the 19 Mucoromycotinian OTU for which over 200 sequences were obtained across samples, all were found in low abundance in the root compartment relative to the bulk soil (Supplementary Table 4). No sequences showed a close match to sequences (Figure 3C) which have been associated with the fine root endophyte mycorrhizal morphology (KX434777 and KX434773, and clustering with KC708398, JF414224, JF414221, and KC708405). However, OTUs from this study formed two distinct clades related to the M-AMF forming clade. Notably OTU114, the most abundant OTU, formed a distinct clade with several other OTUs which appear unrelated to previously published sequences. OTU389 and 236 were related to the putative ectomycorrhizal fungus *Endogone lactiflua*, while OTU55, the second most abundant Mucoromycotinian sequence, showed similarity to the putative saprotroph *Vinositunica radiata*. OTUs 255 and 226 formed a further clade which was distinct from the other Mucoromycotinian sequences.

Overall Mucoromycotinian OTU showed very low relative abundance and diversity within roots, no Mucoromycotinian OTUs showed evidence for enrichment in roots, and phylogenetic analysis of Mucoromycotinian sequences found no evidence for the presence of OTU closely related to those which have previously been shown to form M-AMF. Therefore, Mucoromycotinian sequences were not considered further, and AMF community analysis using 18S rRNA sequences focused on Glomeromycotina only.

Permutational multivariate analysis of variance (Table 4) showed that plantation and compartment accounted for 31.2% (*P* < 0.001) and 3.9% (*P* < 0.001) of Glomeromycotinian community variation, respectively, with pH, P, C, and N contributing to 3.4% (*P* < 0.001), 2.4% (*P* < 0.02), 1.5% (*P* < 0.036), and 1.7% (*P* < 0.010) of Glomeromycotinian community dissimilarity, respectively. NMDS analysis of Glomeromycotinian communities (Figure 4B) showed clear clustering of samples by plantation, but there was only evidence for distinct separation of root samples from bulk soil and rhizosphere soil in the 18- and 35-year old plantations. Most Glomeromycotinian OTUs were specific, or predominantly located, in a single plantation (Figure 4Ci), although a substantial proportion of OTUs were shared between the 18- and 35-year old plantations, but absent in the 2-year old plantation. Most OTUs, including abundant Glomeromycotinian taxa, showed no distinct preference for compartment (Figure 4Cii), occupying the central zone in the ternary plot, although there were a number of low abundance taxa which showed a preference for roots.

**TABLE 4:** Permutational multivariate analysis of variance (PERMANOVA) of the effect of plantation (2-, 18-, and 35-year old locations), compartment (soil vs. rhizosphere soil vs. root) and soil characteristics on Glomeromycotinian fungus community composition.

	DF	SS	MS	*F*.Model	*R* ^2^	Pr(>F)
Plantation	2	8.194	4.097	19.559	0.312	0.001
Compartment	2	1.024	0.512	2.443	0.039	0.002
pH	1	0.887	0.887	4.234	0.034	0.001
Olsen P	1	0.628	0.628	2.999	0.024	0.002
Soil N	1	0.450	0.450	2.146	0.017	0.010
Soil C	1	0.401	0.401	1.912	0.015	0.036
Residuals	70	14.663	0.210		0.559	
Total	78	26.246			1.000	

*Sequencing was performed using 18S rRNA gene primers.*

Similarity percentage analysis identified a number of Glomeromycotinian OTUs which contributed to over 2% dissimilarity between the soil, rhizosphere soil and root compartments (Table 5). Several of these OTUs with relative abundance of over 4% were enriched in the roots compared to the bulk and rhizosphere soils, including OTU3, 8, and 4 which contributed 7.3, 5.1, and 4.0% of the dissimilarity between compartments, respectively.

**TABLE 5:** Similarity percentage (SIMPER) analysis of OTU contributing to arbuscular mycorrhizal fungus community dissimilarity between bulk soil, rhizosphere soil and root compartments.

OTU	% contribution to difference	% relative abundance in bulk soil	% relative abundance in rhizosphere soil	% relative abundance in roots
Glomeromycete OTU3	7.3	6.3	7.8	8.0
Glomeromycete OTU2	5.5	9.4	7.6	3.6
Glomerales OTU10	5.4	6.5	6.2	4.4
Glomeromycete OTU8	5.1	4.6	4.0	5.7
Glomeraceae OTU4	4.0	4.1	4.2	7.0
Glomerales OTU14	3.0	2.9	3.5	3.7
Glomeromycete OTU6	2.7	2.3	3.0	2.7
Glomerales OTU25	2.6	2.0	2.5	2.6
*Acaulospora* sp. OTU11	2.6	2.8	3.8	4.7
Glomeromycete OTU16	2.5	1.2	2.5	3.5
Glomeromycete OTU39	2.4	1.8	2.4	1.9
Glomeraceae OTU17	2.4	3.4	2.8	1.6
*Acaulospora* sp. OTU36	2.2	2.3	1.6	2.3
Glomeromycete OTU96	2.1	1.7	1.7	2.5

*Sequencing was performed using AMF specific 18S rRNA gene primers. Data is combined across 2-, 18-, and 35-year old oil palm sites. OTUs contributing to >2% of community dissimilarity are shown.*

The key OTUs contributing to dissimilarity between plantations (Table 6) included OTU3 (7.8% of dissimilarity) and OTU8 (5.5% of dissimilarity), which were abundant (19.7 and 14.0% relative abundance, respectively) in the 2-year old plantation, but had low relative abundance in the older plantations, and OTU10 (6.1% of dissimilarity) which was abundant (14.9% relative abundance) in the 18-year old plantation and present in low abundance in the other plantations. A number of further OTUs contributed to over 2.3% of the variance between compartments, and were preferentially abundant at specific sites or combinations of sites. Interestingly, Glomeromycotinian OTU identified by SIMPER analysis showed low homology to established AMF genera, and most could not be identified below the class (Glomeromycete) or order (Glomerales) levels.

**TABLE 6:** Similarity percentage (SIMPER) analysis of OTUs contributing to arbuscular mycorrhizal fungus community dissimilarity between 2-, 18-, and 35-year old plantations.

OTU	% contribution to difference	% relative abundance in 2- year old plantation	% relative abundance in 18- year old plantation	% relative abundance in 35- year old plantation
Glomeromycete OTU3	7.8	19.7	0.3	2.0
Glomerales OTU10	6.1	0.1	14.9	2.1
Glomeromycete OTU8	5.5	14.0	0.0	0.2
Glomeromycete OTU2	5.1	10.1	2.2	8.4
Glomeraceae OTU4	4.1	0.1	8.4	6.8
Glomerales OTU14	3.1	0.0	5.2	4.9
Glomeromycete OTU6	2.7	0.2	1.1	6.7
Glomerales OTU25	2.7	0.0	6.8	0.4
*Acaulospora* sp. OTU11	2.5	4.7	1.4	5.3
Glomeromycete OTU16	2.4	0.3	1.2	5.8
Glomeromycete OTU39	2.4	0.0	0.1	6.0
Glomeraceae OTU17	2.3	0.1	4.4	3.4

*Bulk soil, rhizosphere soil, and root compartments are pooled within each site. Sequencing was performed using AMF specific 18S rRNA gene primers. OTU contributing to >2% of AMF community dissimilarity are shown.*

## Discussion

Despite the considerable importance of palm oil as a global commodity and the widespread cultivation of oil palm across SouthEast Asia, South America and Africa, little is known about the composition or function of its microbiome. The root associated fungal microbiome across all sites was enriched in Agaricomycetes, Glomeromycetes and Lecanoromycetes relative to the bulk soil. Despite the close geographical proximity of sampling sites, for both fungal (ITS) and Glomeromycotinian (18S rRNA) communities, plantation had the dominant effect on community variation, with the combined contributions of soil properties more important than compartment, particularly for Glomeromycotinian communities. A number of putative pathogens were found in roots and soil across all 3 sites, notably taxa related to *Prospodicola mexicana* and *Pleurostoma* sp. No evidence was found for enrichment of Mucoromycotinian fungi in roots, or the presence of sequences previously shown to form fine root endophyte arbuscular mycorrhizas, suggesting the AMF community across the plantations was comprised of Glomeromycotinian fungi only. While there was evidence for selection of Glomeromycotinian communities in oil palm roots, this was a weak effect, and abundant taxa dominated across root, rhizosphere soil and bulk soil compartments. Generally, dominant fungal sequences, including the Glomeromycotina and Mucoromycotina, showed low similarity to described sequences, suggesting there was considerable novel soil biodiversity with significance for plant health within the plantations.

Due to primer mismatches, the ITS2 region provides poor coverage of Glomeromycotinian diversity, and use of 18S rRNA provides a more comprehensive assessment of diversity. However, ITS2 and 18S rRNA have been shown to provide similar resolution of broad Glomeromycotina diversity patterns in root and soil compartments (Berruti et al., 2017). In our study, ITS amplicon analysis showed increased relative abundance of Glomeromycotina OTUs in the roots relative to the soil and rhizosphere compartments only in the 35-year old plantation, but there was no evidence that the root and soil compartments supported different Glomeromycotina communities. The 18S rRNA amplicon analysis showed a significant effect of compartment on community composition, and there was evidence for differences in Glomeromycotina community composition between root and soil compartments in the 18- and 35-year old plantations, but not in the 2-year old plantation.

In contrast to Berruti et al. (2017), our data suggests that ITS2 primers lacked the power to discriminate differences in Glomeromycotina community structure between roots and soil relative to AMF specific 18S rRNA primers. This highlights potential problems when using low sequencing depth data from broad fungal community sequencing studies using ITS2 to inform on the structure of Glomeromycotina communities, and small but biologically significant differences in community structure and environmental interactions may be missed.

Glomeromycotina sequences were detected in the root compartment of oil palm in all plantations, suggesting the presence of the arbuscular symbiosis within palm roots. Currently, M-AMF forming the fine root endophyte arbuscular morphology has been linked to a small clade within the Endogolales, centered on OTU0 and OTU4 from Orchard et al. (2017), the latter showing 100% sequence match to the only described species of M-AMF fine root endophyte, *Planticonsortium tenue* (Albornoz et al., 2021). Albornoz et al. (2020a,b) found FRE colonization in 44 of 58 Australian pastures sampled, and 92% of the associated Mucoromycotinian sequences clustered within this clade, with 74% of sequences belonging to three OTUs which were distinct from both OTU0 and 4. While we detected diverse Mucoromycotinian OTUs across the plantations, none fell within the clade comprising putative M-AMF forming taxa, and all the Mucoromycotinian OTUs were rarely detected in roots. Overall the evidence suggests that M-AMF were not present on oil palm roots. In a study of Australian biomes, Albornoz et al. (2021) found that across 10 Australian biomes putative M-AMF were favored by agricultural land use, were rare or absent in native ecosystems, but were not found in tropical biomes.

Our work has show that plantation was the dominant factor controlling both fungal (ITS) and Glomeromycotinian community variation across samples. This could result from a wide range of factors including age, management, and prior land use. Plantation exerted greater effects on Glomeromycotinian community composition relative to the fungal (ITS) community, and similarly the combined contribution of soil properties to Glomeromycotinian community variation was greater than for fungal (ITS) communities. This may reflect the dependence of AMF communities on plant nutritional requirements, and may therefore be associated with differences in tree age and nutrient management differences across plantations. However, it was surprising that the obligate symbiont Glomeromycotan communities were less strongly determined by compartment than were fungal (ITS) communities.

The small effect of compartment on Glomeromycotinian community composition in the 18S rRNA analysis indicates weak selection of communities by plant roots, and this only occurred in the older plantations. Furthermore a recent study in Amazonia reported low rates of AMF colonization (5–20%) in tertiary and quaternary oil palm roots when grown in monoculture relative to when grown in mixed species agroforestry plantations (20–50%). Palm roots are long lived and often lignified, making observation of AMF colonization difficult (Fisher and Jayachandran, 2005). As a result the mycorrhizal status of palm species in the field has rarely been determined, and most studies have focused on seedlings (Fisher and Jayachandran, 1999, 2005). While palms are typically mycorrhizal, species may vary in the intensity of AM colonization and the morphology of fungal structures formed within roots (Fisher and Jayachandran, 2005). Jourdan et al. (2000) identified 8 distinct types of primary and secondary roots in oil palm, based on a number of parameters including gravitropism, secondary thickening and length. In the palm *Serenoa repens*, arbuscular mycorrhizal structures were seen in all root orders, but were most frequent in the fine, higher order roots (Fisher and Jayachandran, 1999).

Our collection method did not discriminate between fine and coarse oil palm roots, and specific focus on fine roots may provide greater clarity on the mycorrhizal status of mature oil palm, including evidence for root colonization and the presence of arbuscules. Violita et al. (2016) showed seasonal variation in oil palm fine root biomass in a Malaysian plantation, with fine root density in March almost half that in December, June and September. This variation was associated with changes in temperature. Mycorrhizal communities could therefore show variation in time, reflecting abundance of fine root biomass, suggesting a need to characterize seasonal dynamics of AMF communities to understand the importance of the arbuscular symbiosis in oil palm. A further factor which could account for low filtering of Glomeromycotinian communities by compartment is low soil disturbance in plantations. This could result in the development of a stable and persistent community, with abundant taxa in the system dominating across roots, rhizosphere soil and bulk soil.

Many authors have identified AMF as a component of the oil palm microbiome which could contribute to sustainability through inoculation of strains with enhanced nutrient scavenging or pathogen control potential (Phosri et al., 2010; Sundram et al., 2015). Even in temperate agricultural systems in which AMF-plant interactions have been intensively studied, there is limited direct evidence linking AMF abundance and diversity to crop health and yield (Ryan and Graham, 2018), although there is recognition that AMF have the potential to contribute to system sustainability in ways other than promoting yield, such as contributing to reduced fertilizer inputs, and promoting soil structure (Rillig et al., 2019). Benefits of AMF on crop yield, nutrition and system sustainability are likely to vary considerably depending on crop species and variety, soil characteristics and agronomic management practices, and there is a clear need for future research to untangle these interactions in oil palm systems.

The best studied pathogen associated with oil palm is basal stem rot, *Ganoderma boninense*, which is widespread in oil palm in SouthEast Asia (Hushiarian et al., 2013) and is particularly prevalent in replanted areas. In Africa and South America *Fusarium* spp., particularly *F. oxysporum* f. sp. *elaeidis* is a problematic oil palm pathogen, and a number of other fungal pathogens have been identified in oil palm leaves and roots, including *Thielaviopsis paradoxa*, *Pestalotiopsis microspora*, and *Curvularia affinis* (Paterson et al., 2013), although the effect of these fungi on host health is unclear.

None of the aforementioned fungal pathogens were present in our study, but FUNGuild analysis identified the presence of putative fungal plant pathogens across the plantations. This is likely to be an underestimate as many of the ITS fungal sequences were not closely related to known taxa, and could not be assigned a guild, including OTU6, which was highly abundant in roots, and most closely related to the pathogen *Pleurostoma richardsiae* which is implicated in decline of olive trees (Lawrence et al., 2020). A number of putative pathogens identified by FUNGuild were enriched in the roots relative to the bulk soil and rhizosphere. By far the most abundant of these was OTU8 which showed close similarity to *Prosopidicola mexicana*. This OTU was one of the most abundant fungi detected in the roots in all three plantations, comprising 3.9–7.4% of fungal community relative abundance. *P. mexicana* causes pod disease in *Prosopis* sp. leguminous shrubs, and has been proposed as biocontrol agent of invasive plant species (Lennox et al., 2004). The other putative pathogens were present in low abundance, including Magnaporthaceae OTU74, *Acrophialophora levis* OTU78, *Veronaea botryosa* OTU142 and *Cylindrosympodium* sp. OTU150 which were detected in roots. The Magnaporthaceae family includes fungal species that cause devastating diseases on cereals and grasses (Illana et al., 2013), while *A. levis* has been shown to cause wilt in *Plumeria acutifolia* shrubs (Kumar et al., 2016). *Veronaea botryose and Cylindrosympodium* are better known as rare opportunistic pathogens of humans (Crous et al., 2007). As well as including exclusively root-residing pathogens, root fungal communities may also include foliar pathogens which can grow from the shoots into the root (Picot et al., 2021). Therefore the putative root associated pathogens we detected could potentially be linked with both foliar and root diseases.

A number of further putative plant pathogens were detected in plantation soil including OTU36 (*Plectosphaerella oligotrophica*), OTU98 (*Triparticular* sp.), OTU141 (*Plectosphaerella delsorboi*) and OTU125 (*Diatrypella atlantica). Plectosphaerella* sp. are pathogens of a wide range of plants, causing fruit, root and collar rot (Su et al., 2017). Although *Diatrypella* spp. are generally considered to be saprophytes, they may be asymptomatic or hemibiotrophic associates of plants, and have been identified as pathogens of grapevines (Pitt et al., 2013). The presence of these putative pathogens in the soil could reflect occurrence in the canopy as foliar pathogens, or localized presence on unsampled tree and understory roots within the plantations.

Fungi that we found associated with oil palm roots likely represent fungal endopytes. A range of fungal endophytes have been identified in oil palm leaves including *Pycnoporus* sp., *Pyrenochaetopsis* sp., and in common with our study, *Fomitopsis* sp. (Pinruan et al., 2010; de Assis Costa et al., 2018). de Assis Costa et al. (2018) studied fungal communities of oil palm leaves across developmental stages of Fatal Yellowing disease of oil palm in South America, the cause of which is still unknown. While several putative pathogens were detected in symptomatic samples, including *Colletotrichum* sp. and *Fusarium* sp., the composition of these communities changed with disease developmental stage, and they are likely to represent secondary infections, with their effects on plant health unclear.

Interestingly oil palm roots and soil in the young oil palm plantation had greater relative abundance of putative pathogens, and lower relative abundance of AMF than the older plantations. A range of factors could contribute to differences in fungal community composition between the sites, including plantation age, fertilization strategies, prior land use and crop genotype differences. Further research is required to understand the relative contribution of these different factors to determining the assembly of the oil palm microbiome.

Notably, many of the abundant fungal sequences, including the Glomeromycotina and Mucoromycotina, showed low similarity to existing sequences, suggesting considerable novel biodiversity in the plantations. However, some of the fungi we detected may have widespread distribution across plant roots, particularly in tropical regions, suggesting important ecological interactions. For example, OTU3 matched sequences found in roots in Costa Rica (Urbina et al., 2016) and the Netherlands (Hannula et al., 2012), and OTU4 matched a sequence found in *Allanblackia stuhlmannii* roots in Tanzania (Kartzinel et al., 2013). Intriguingly, the closest match for OTU6 was found in the same *Allanblackia* root sample which contained the sequence match for OTU4, which could suggest an ecological relationship between these fungi.

Generally, global understanding of soil biodiversity is limited and less comprehensive than that of aboveground biodiversity. Similarly, knowledge of the ecosystem functions of soil biota is sparse and largely focused on temperate regions, and the degree to which this knowledge can be extended to tropical areas is unclear (Guerra et al., 2020). Plant health is determined by complex interactions with diverse biota, including mutualists and pathogens. Our study has identified a range of fungal taxa which dominated the oil palm root fungal microbiome. Several of the most abundant fungi were most closely related to pathogens, and the microbiome also included Glomeromycotinian arbuscular fungi. However, since most root associated fungi represented uncultured taxa the way in which they interact with the host and influence its health is unclear. Targeted analysis of these taxa, including isolation, will be important to understand the contributions of these fungi to plant health, and this may facilitate strategies for the sustainable management of the oil palm microbiome.

## Data Availability Statement

The datasets presented in this study can be found in online repositories. The names of the repository/repositories and accession number(s) can be found below: https://www.ncbi.nlm.nih.gov/, PRJNA755964 and PRJNA756009.

## Author Contributions

GB, NM, GO, and AS: project conception, project funding, and experimental design. GB, NM, GO, AS, GS, AT, JC, and DE: field sampling, sample processing, and DNA extraction. GS and DE: soil analysis. SH: sequencing. GB, SH, and EK: bioinformatic and statistical analysis. DB: fungal phylogenetic analysis. GB and EK: writing the manuscript. All authors edited the manuscript.

## Conflict of Interest

The authors declare that the research was conducted in the absence of any commercial or financial relationships that could be construed as a potential conflict of interest.

## Publisher’s Note

All claims expressed in this article are solely those of the authors and do not necessarily represent those of their affiliated organizations, or those of the publisher, the editors and the reviewers. Any product that may be evaluated in this article, or claim that may be made by its manufacturer, is not guaranteed or endorsed by the publisher.
