# Supplementary material for: Diversity and Ecological Guild Analysis of the Oil Palm Fungal Microbiome Across Root, Rhizosphere, and Soil Compartments
Source: Front Microbiol. 2022 Feb 11;13:792928. doi: 10.3389/fmicb.2022.792928 (PMC8874247; doi:10.3389/fmicb.2022.792928)
Supplement: Supplementary file 1 [file Data_Sheet_1.docx]

**Table S1 Permutational multivariate analysis of variance (PERMANOVA) of the effect of plantation (2-, 18- and 35-year old locations), compartment (soil vs rhizosphere soil vs root) and soil characteristics on arbuscular fungus (AMF) community composition.** Data represents OTU identified as highly probable and probable AMF using the FUNGuild annotation tool, using fungal ITS sequences.

|  | **DF** | **SS** | **MS** | **F.Model** | **R^2^** | **Pr(>F)** |
| --- | --- | --- | --- | --- | --- | --- |
| **Plantation** | **2** | **7.650** | **3.825** | **12.968** | **0.240** | **0.001** |
| **pH** | **1** | **0.812** | **0.812** | **2.754** | **0.026** | **0.003** |
| **Olsen P** | **1** | **0.720** | **0.721** | **2.443** | **0.023** | **0.003** |
| **Soil C** | **1** | **0.607** | **0.607** | **2.057** | **0.019** | **0.014** |
| **Soil N** | **1** | **0.620** | **0.620** | **2.102** | **0.019** | **0.005** |
| Compartment | 2 | 0.793 | 0.398 | 1.345 | 0.025 | 0.075 |
| Residuals | 70 | 20.647 | 0.295 |  | 0.648 |  |
| Total | 78 | 31.850 |  |  | 1.000 |  |

Bold indicates a significant effect

**Table S2 Permutational multivariate analysis of variance (PERMANOVA) of the effect of plantation (2-, 18- and 35-year old locations), compartment (soil vs rhizosphere soil vs root) and soil characteristics on fungal pathogen community composition.** Data represents OTU identified as highly probable and probable plant pathogens using the FUNGuild annotation tool, using fungal ITS sequences.

|  | **DF** | **SS** | **MS** | **F.Model** | **R^2^** | **Pr(>F)** |
| --- | --- | --- | --- | --- | --- | --- |
| **Plantation** | **2** | **5.429** | **2.714** | **9.564** | **0.182** | **0.001** |
| **Compartment** | **1** | **2.081** | **1.040** | **3.665** | **0.070** | **0.001** |
| **Soil C** | **2** | **0.637** | **0.637** | **2.243** | **0.021** | **0.002** |
| **Soil N** | **1** | **0.567** | **0.587** | **2.066** | **0.020** | **0.003** |
| **pH** | **1** | **0.493** | **0.493** | **1.736** | **0.017** | **0.019** |
| Olsen P | 1 | 0.394 | 0.394 | 1.387 | 0.013 | 0.094 |
| Residuals | 71 | 20.152 | 0.284 |  | 0.677 |  |
| Total | 79 | 29.771 |  |  | 1.000 |  |

Bold indicates a significant effect

**Table S3 Analysis of similarity percentage (SIMPER) of differences in fungal plant pathogen OTU between oil palm bulk soil and root compartments.** Data is combined across 2-, 18- and 35-year old oil palm sites. Data represents OTU identified as highly probable and probable plant pathogens using the FUNGuild annotation tool, using fungal ITS sequences. OTU contributing to >than 2 % of plant pathogen community dissimilarity are shown.

| **OTU** | **% contribution to difference** | **% relative abundance in bulk soil** | **% relative abundance in root** |
| --- | --- | --- | --- |
| *Prosopidicola mexicana* OTU8 | 36.7 | 0.2 | 5.3 |
| *Plectosphaerella oligotrophica* OTU36 | 6.1 | 0.4 | 0.3 |
| Magnaporthaceae OTU74 | 5.6 | 0.3 | 0.7 |
| *Acrophialophora levis* OTU78 | 5.0 | 0.2 | 0.7 |
| *Triparticalcar* sp. OTU98 | 4.9 | 0.4 | 0.0 |
| *Plectosphaerella delsorboi* OTU141 | 3.6 | 0.5 | 0.1 |
| *Diatrypella atlantica* OTU125 | 2.9 | 0.2 | 0.1 |
| *Rhizophydium* sp. OTU139 | 2.6 | 0.2 | 0.0 |
| *Veronaea botryosa* OTU142 | 2.4 | 0.0 | 0.2 |
| *Cylindrosympodium* sp. OTU150 | 2.7 | 0.0 | 0.2 |
| *Lasiodiplodia theobromae* OTU122 | 2.0 | 0.1 | 0.1 |

**Table S4 Abundance of Mucoromycotinian OTU in bulk soil, rhizosphere soil and root compartments.** Each data point represents the number of sequences detected within the pool of 270K rarefied (Glomeromycotinian and Mucoromycotinian) sequences used for each compartment type, combined across plantations. All OTUs for which more than 200 sequences were detected across plantations and compartments are shown. Phylogenetic relatedness of each OTU is shown in Figure 3. Sequencing was performed with 18S rRNA gene primers.

| **OTU** | **Total number of sequences** | | |
| --- | --- | --- | --- |
|  | **Bulk Soil** | **Rhizosphere soil** | **Roots** |
| 55 | 1274 | 1036 | 65 |
| 88 | 851 | 625 | 50 |
| 95 | 1343 | 716 | 11 |
| 104 | 640 | 404 | 48 |
| 114 | 1673 | 768 | 7 |
| 133 | 735 | 328 | 6 |
| 137 | 401 | 232 | 14 |
| 148 | 403 | 227 | 1 |
| 180 | 446 | 252 | 5 |
| 182 | 120 | 118 | 13 |
| 188 | 56 | 149 | 50 |
| 226 | 180 | 85 | 4 |
| 236 | 225 | 47 | 0 |
| 255 | 138 | 106 | 3 |
| 389 | 141 | 80 | 32 |
| 505 | 175 | 38 | 6 |
| 535 | 127 | 120 | 65 |
| 1539 | 267 | 113 | 4 |
