# Supplementary material for: Diversity and Ecological Guild Analysis of the Oil Palm Fungal Microbiome Across Root, Rhizosphere, and Soil Compartments
Source: Front Microbiol. 2022 Feb 11;13:792928. doi: 10.3389/fmicb.2022.792928 (PMC8874247; doi:10.3389/fmicb.2022.792928)

Figure S1 Fungal community rarefaction curves (a). ITS primers (b). 18S rRNA gene primers

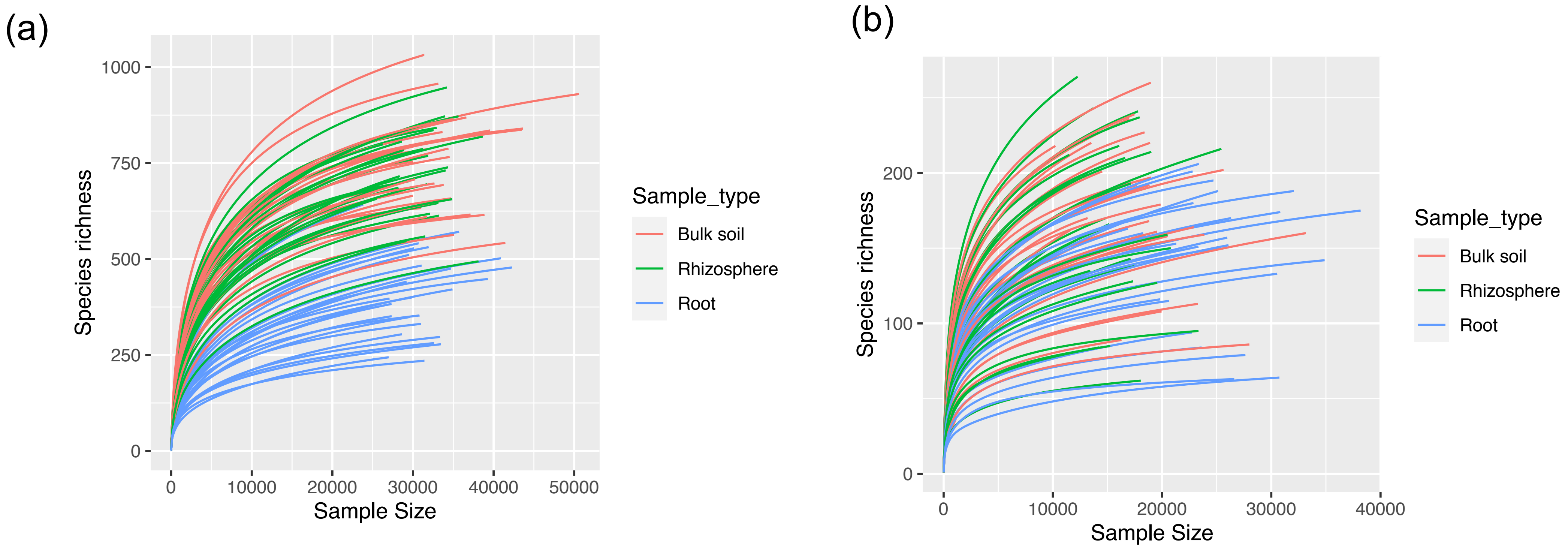

Figure S2. Soil characteristics in 2-, 18- and 35-years old oil palm plantations a) total carbon, (b) total nitrogen, (c) pH and (d) Olsen-P. Bars with different letters are significantly different (P<0.05). Error bars represent +/- standard error of the mean

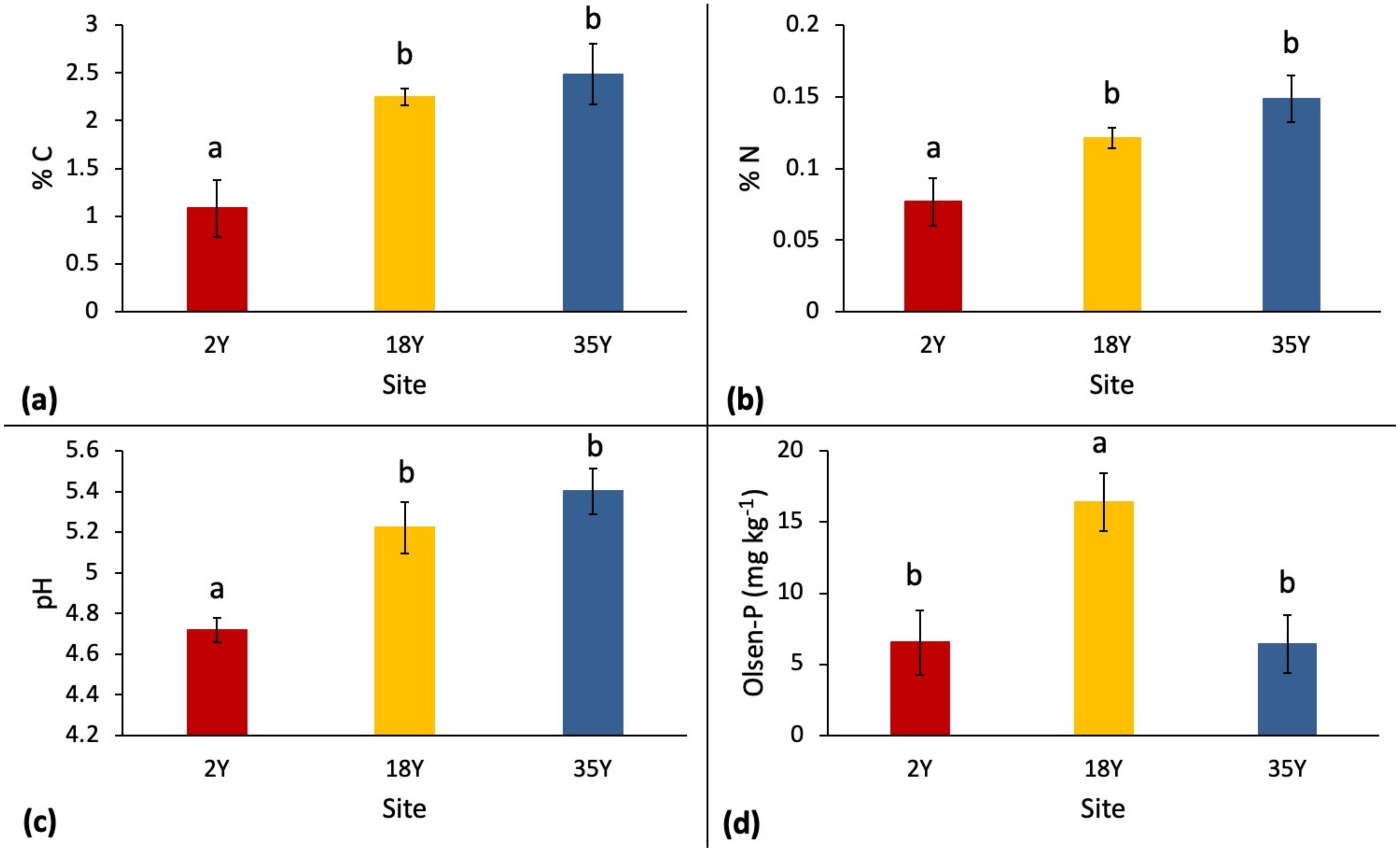

Figure S3. Phylogenetic analysis of fungus ITS gene sequences showing most closely related taxa to OTUs 0, 3, 4, 6 and 8

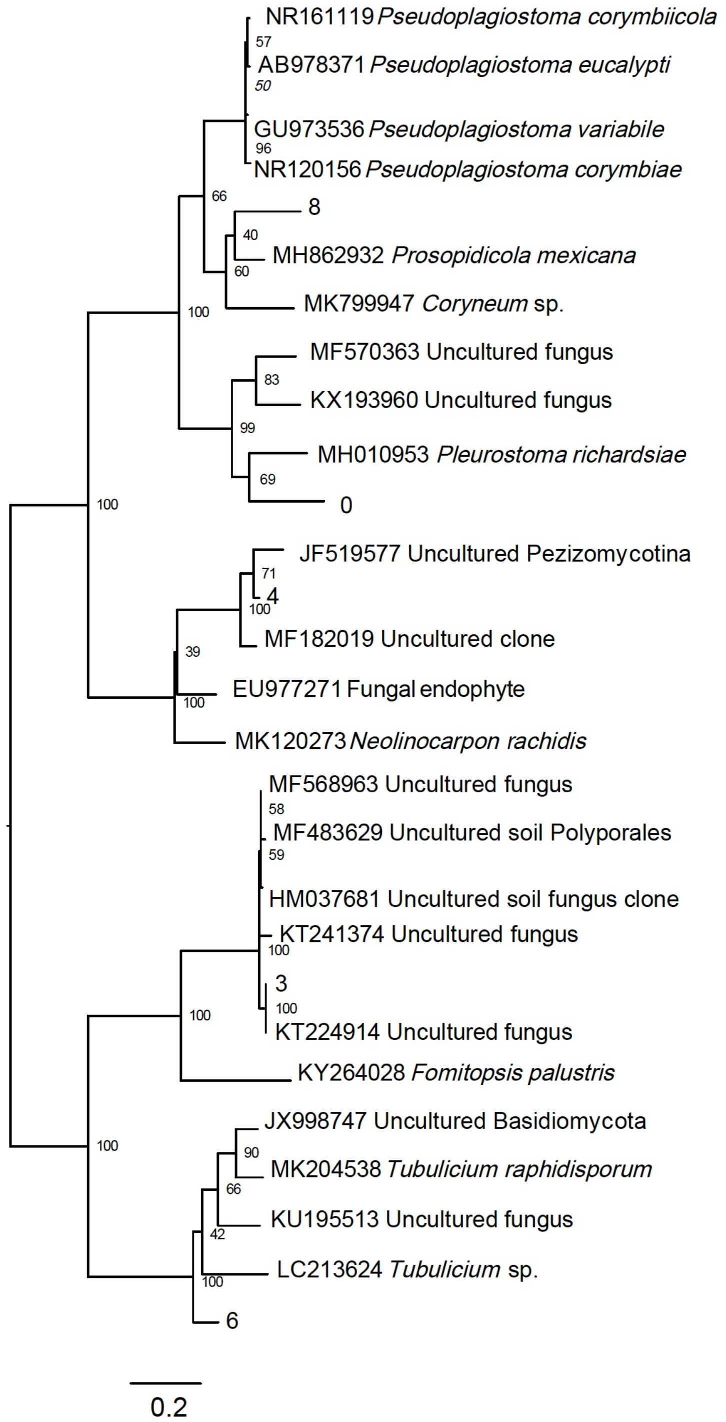

Figure S4. Fungal ecological guilds analysis of ITS data. A. Guilds in bulk soil, rhizosphere soil and root compartments; i) tropic mode ii). pathotroph guilds and iii). symbiotroph guilds. Data is combined across 2-, 18- and 35-year old plantations. B. Guilds in 2-, 18- and 35-year old plantations i). trophic mode ii). pathotroph guilds iii) symbiotroph guilds. Data is combined across root, rhizosphere soil and bulk soil for each plantation. AMF: Arbuscular mycorrhizal fungus, EcM: Ectomycorrhizal fungus. Sequencing was performed with ITS primers. Bars with different letters are significantly different ($p<0.05$). Error bars represent +/- standard error of the mean

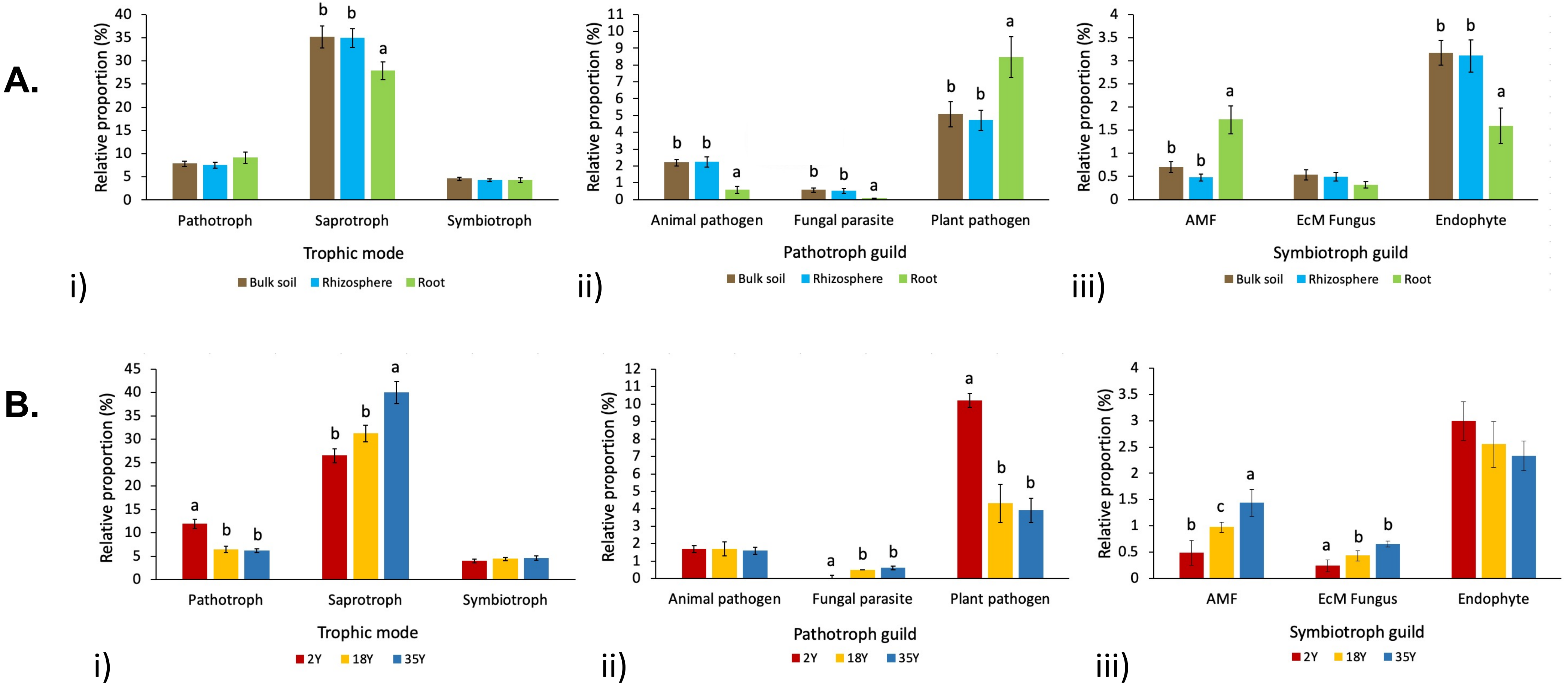

Fig S5. Relative abundance of A. Arbuscular mycorrhizal fungal sequences and B. Plant pathogen sequences in i). 2-year old plantation ii). 18-year old plantation iii). 35-year old plantation. Data includes only ITS OTU annotated as highly probable and probable. Sequencing was performed with 18SrRNA gene primers. Bars with different letters are significantly different ($p<0.05$).

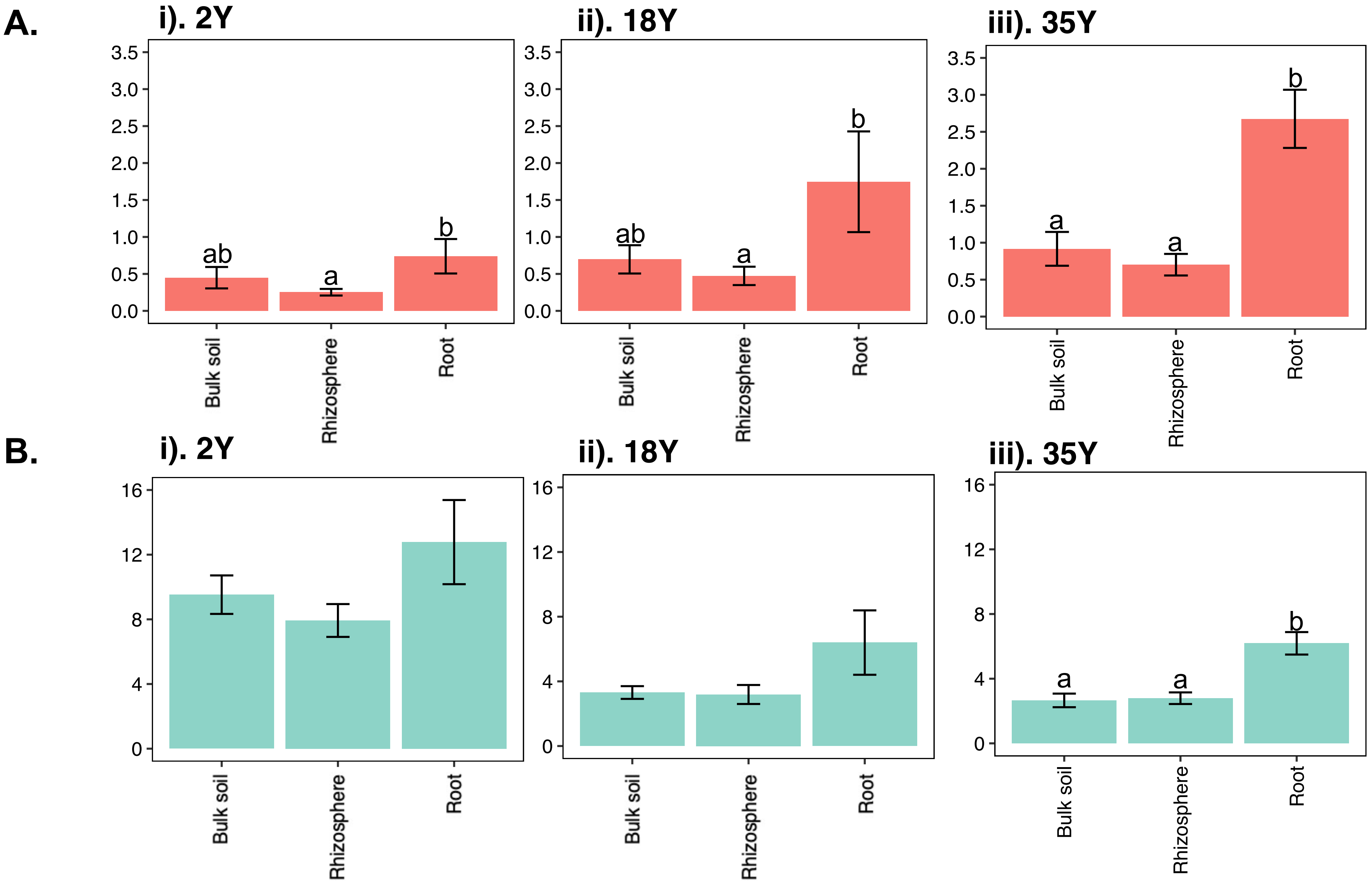

Figure S6. Non-metric multidimensional scaling analysis of Bray Curtis dissimilarity of A. arbuscular mycorrhizal fungus and B. plant pathogen communities in bulk soil, rhizosphere soil and roots of 2-, 18- and 35-year old oil palm plantations. Sequencing was performed with ITS primers. Data includes only OTU annotated as highly probable and probable AMF or plant pathogens.

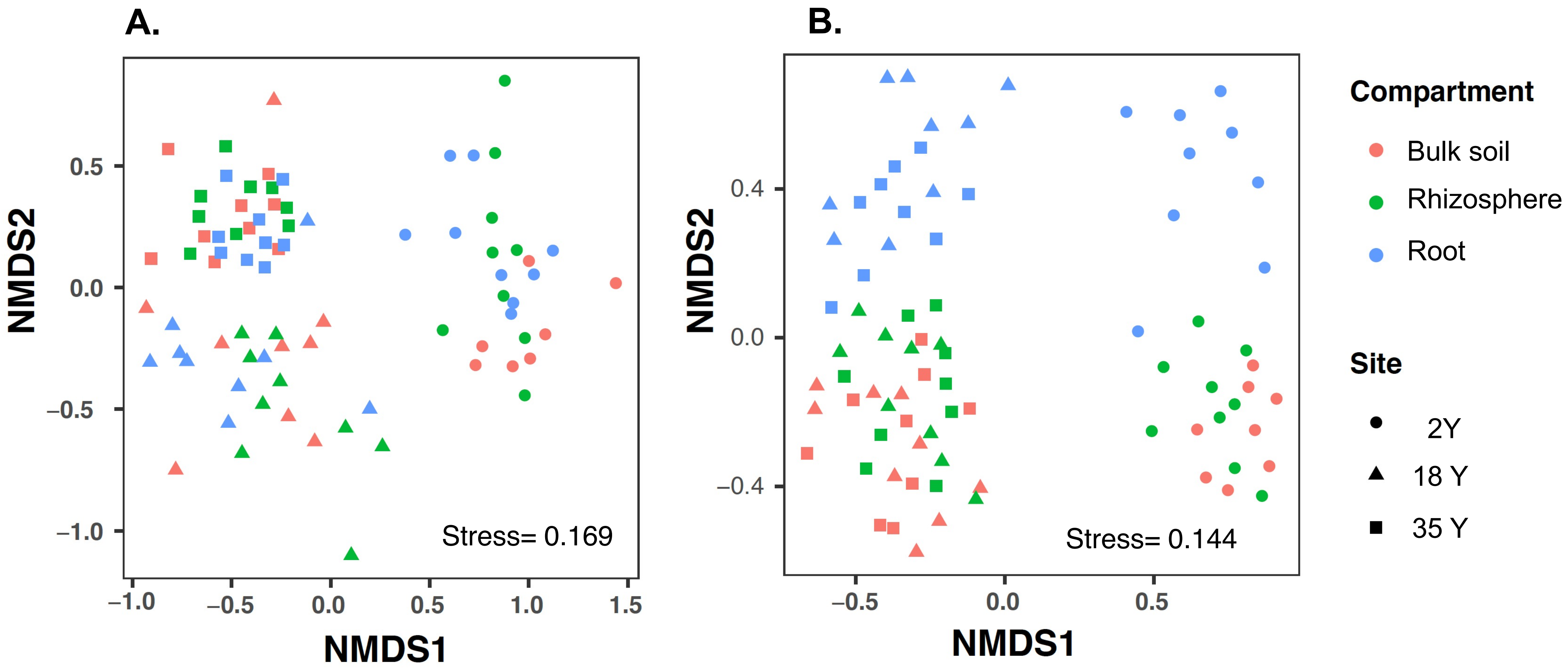

Supplement: Supplementary file 2 [file Presentation_1.pdf]
